# *IL12RB2* Gene Is Associated with the Age of Type 1 Diabetes Onset in Croatian Family Trios

**DOI:** 10.1371/journal.pone.0049133

**Published:** 2012-11-13

**Authors:** Marina Pehlić, Dina Vrkić, Veselin Škrabić, Ana Jerončić, Gordana Stipančić, Anita Špehar Urojić, Igor Marjanac, Jasminka Jakšić, Zrinka Kačić, Vesna Boraska, Tatijana Zemunik

**Affiliations:** 1 Department of Medical Biology, University of Split, School of Medicine, Split, Croatia; 2 Croatian Centre for Global Health, University of Split, School of Medicine, Split, Croatia; 3 Department of Pediatrics, University Hospital Center Split, Split, Croatia; 4 Department of Research in Biomedicine and Health, University of Split, School of Medicine, Split, Croatia; 5 Department of Pediatrics, University Hospital “Sestre Milosrdnice” Zagreb, Zagreb, Croatia; 6 Department of Pediatrics, University Hospital Center Rebro, Zagreb, Croatia; 7 Department of Pediatrics, University Hospital Center Osijek, Osijek, Croatia; 8 Department of Pediatrics, General Hospital, Šibenik, Croatia; 9 Department of Pediatrics, General Hospital Dubrovnik, Dubrovnik, Croatia; University of Michigan Medical School, United States of America

## Abstract

**Background:**

Common complex diseases are influenced by both genetic and environmental factors. Many genetic factors overlap between various autoimmune diseases. The aim of the present study is to determine whether four genetic variants known to be risk variants for several autoimmune diseases could be associated with an increased susceptibility to type 1 diabetes mellitus.

**Methods and Findings:**

We genotyped four genetic variants (rs2358817, rs1049550, rs6679356, rs9865818) within *VTCN1, ANXA11, IL12RB2* and *LPP* genes respectively, in 265 T1DM family trios in Croatian population. We did not detect association of these polymorphisms with T1DM. However, quantitative transmission disequilibrium test (QTDT, orthogonal model) revealed a significant association between the age of onset of T1DM and *IL12RB2* rs6679356 variant. An earlier onset of T1DM was associated with the rs6679356 minor dominant allele C (p = 0.005). The association remained significant even after the Bonferroni correction for multiple testing and permutation.

**Conclusions:**

Variants originally associated with juvenile idiopathic arthritis (*VTCN1* gene), sarcoidosis (*ANXA11* gene), primary biliary cirrhosis (*IL12RB2* gene) and celiac disease (*LPP* gene) were not associated with type 1 diabetes in our dataset. Nevertheless, association of *IL12RB2* rs6679356 polymorphism with the age of T1DM onset suggests that this gene plays a role in defining the time of disease onset.

## Introduction

Type 1 diabetes mellitus (T1DM) is a chronic autoimmune disease where both genetic and environmental factors play important roles [1]. In the past few years, genome-wide association studies (GWAS) have become dominant in the research of genetic background of various complex diseases, including T1DM [2]. The results of these studies, as well as meta-analyses that followed, confirmed around 50 T1DM susceptibility loci [3], [4]. Numerous T1DM associated genetic loci show an overlap with other immune-mediated diseases [5], [6], [7]. Genes that were shown to be common in the pathogenesis of autoimmune diseases are those that encode pro-inflammatory mediators such as cytokines, antigen processing and presenting molecules, T cell activation pathway molecules, cell adhesion molecules and molecules related to natural killer cells that mediate cytotoxicity [8], [9].

Hinks et al. showed strong association of markers within the V-set domain containing the T cell activation inhibitor 1 (*VTCN1*) gene with juvenile idiopathic arthritis (JIA). *VTCN1*, also known as *B7H4* gene, is involved in inhibitory pathways and is important in the prevention of inflammatory response [10]. The first GWAS study of sarcoidosis identified common nonsynonymous SNP (rs1049550) within the annexin 11 (*ANXA 11)* regulator of cell apopoptosis gene to be strongly associated with the disease [11]. Genetic research of primary biliary cirrhosis (PBC) showed significant association of rs6679356 within the interleukin 12 receptor, beta 2 (*IL12RB2)* gene. The protein encoded by *IL12RB2* gene is a type I transmembrane protein identified as a subunit of the interleukin 12 receptor complex [12]. Recent GWAS of celiac disease (CD) identified 3q28 region, harbouring LIM domain containing preferred translocation partner in lipoma *(LPP)* gene, a gene that codes a transcriptional co-activator protein that has a role in cell to cell adhesion [13].

T1DM is considered to be childhood and adolescent disease with two peaks of onset; one between ages 5 and 9 and other between ages 10 and 14 [14], [15]. The aim of the present study was to test the association of four established autoimmune disease risk polymorphisms with T1DM and to analyse their association with the T1DM age of onset.

## Materials and Methods

### Ethics Statement

This study was approved by the Ethics Committee of the University of Split, School of Medicine, Soltanska 2, 21000 Split in accordance with the Declaration of Helsinki. Written informed consent was acquired from parents or legal guardians before blood sampling.

### Subjects

Blood samples of individuals from 265 parent-offspring trio families (total of 795 samples) were collected at Pediatric Units at several Medical Centres in Croatia. T1DM diagnostics followed the World Health Organization criteria (http://www.who.int/diabetes/publications/Definition%20and%20diagnosis%20of%20diabetes_new.pdf). There were 131 (50.56%) girls and 134 (49.43%) boys among T1DM cases. The mean age of T1DM onset was 8.42 (±4.24) years (SD). There were 92 (34.8%) other family members with T1DM and/or T2DM. Overall, 29 (10.98%) affected children were also diagnosed with an additional autoimmune disease: 15 (51.7%) with autoimmune thyreoiditis, 5 (17.2%) with asthma and 3 (10.3%) with CD.

### Genotyping and SNP Selection

We extracted genomic DNA from peripheral blood leukocytes using the QIAamp DNA Blood Mini Kit (Qiagen, Hilden, Germany) kit. Genotyping of four SNPs rs2358817, rs1049550, rs6679356 and rs9865818 within *VTCN1,ANXA11, IL12RB* and *LPP* genes, respectively, were performed by real-time PCR using ABIPRISM 7500 Sequence Detection System (Applied Biosystems, Foster City, USA) and pre-developed TaqMan assay reagents, C_16212775_10 for rs2358817 SNP, C_7881261_1 for rs1049550 SNP, C_29129912_10 for rs6679356 SNP, and C_11795341_10 for rs9865818 SNP. PCR reaction was carried out according to the manufacturer’s protocol.

### Statistical Analysis

Quality control (QC) of the obtained genotypes was performed prior to association analysis. We tested Mendelian inheritance, Hardy–Weinberg equilibrium (HWE) and minor allele frequencies (MAF) using Plink (http://pngu.mgh.harvard.edu/~purcell/plink/) [16]. MAFs of four SNPs were compared with the National Center for Biotechnology Information SNP database (NCBI dbSNP) MAFs for the central European (CEU) population (www.ncbi.nlm.nih.gov/projects/SNP/). Familybased analyses were carried out using implementations of the transmission disequilibrium test (TDT) in Plink. The association with the age of T1DM onset was performed using quantitative transmission disequilibrium test–Abecasis’s orthogonal association model, implemented in statistical software QTDT (Version 2.4.5 http://www.sph.umich.edu/csg/abecasis/QTDT/) [17]. Evidence for association was assessed using the age of onset as a continuous variable under additive and dominant genetic models. Power calculation was performed using Quanto [18]. After Bonferroni correction for multiple testing, p-values less than 0.0125 were considered nominally significant. In addition, empirical p-values were estimated by Abecasis’s orthogonal test option of the QTDT software, using 10 000 Monte Carlo permutations. The empirical significance threshold of 0.05 corresponded to QTDT p-value of <0.0128.

## Results

The QC analysis found no discrepancies in Mendelian inheritance. The distribution of genotypes for rs9865818 slightly deviated from HWE in healthy parents (P = 0.002). There were no deviations in the expected allele transmissions of 4 investigated SNPs (Table 1) from parents to affected child. Our study had 80% statistical power to detect (at α = 0.0125) an effect of [OR] = 1.98 for rs2358817, [OR] = 1.54 for rs1049550, [OR] = 1.60 for rs6679356 and [OR] = 1.95 for rs9865818, assuming an additive model.

**Table 1:** Transmission disequilibrium analysis for *IL12RB, ANXA11, VTCN1* and *LPP* gene SNPs in 265 family trios (T:U – copies of the minor allele transmitted (T) and untransmitted (U) from heterozygous parents to affected offspring*).*

SNP	Alleles (T:U)	T:U	OR	95% CI	*χ2*	*p*-value*
rs2358817	T:C	24∶20	1.2	0.6629–2.172	0.3636	0.5465
rs1049550	G:A	126∶132	0.9545	0.7478–1.218	0.1395	0.7087
rs6679356	T:C	94∶92	1.022	0.7665–1.362	0.0215	0.8834
rs9865818	G:A	118∶110	1.073	0.8273–1.391	0.2807	0.5962

* Nominal p values are shown uncorrected for multiple testing. The Bonferroni-corrected significance threshold is p = 0.0125.

The QTDT analysis detected significant association of the *IL12RB2* rs6679356 variant with the T1DM age of onset (Table 2). The association was significant in both additive and dominant genetic models, but the dominant model produced a better fit (R^2^ 7% vs 4%), indicating allele C as the dominant allele. Individuals with the risk allele C (homozygotes for the C allele and heterozygotes) had a median age of onset 13 months earlier (median 88 months, IQR 49–120) than homozygous for the non-risk allele T (101 month, IQR 63–141). The Bonferroni correction and the permutation tests further confirmed the observed association of the 4 gene variants with the early-onset of T1DM (Table 2).

**Table 2:** Quantitative transmission disequilibrium analysis (Abecasis’s orthogonal test) in 262 family trios with age of T1DM onset as a quantitative variable.

SNP	Minor allele	*p*-value*	Empirical *p*-value**	Optimal Genetic Model
rs2358817	T	0.782	0.778	Additive
rs1049550	A	0.098	0.144	Additive
rs6679356	C	0.005	0.003	Dominant
rs9865818	G	0.544	0.541	Additive

* Nominal p values are shown uncorrected for multiple testing. The Bonferroni-corrected significance threshold was p = 0.0125.

** Empirical p-values were obtained using 10000 Monte Carlo permutations. Empirical significance threshold of 0.05 corresponded to a p-value of <0.0128.

## Discussion

Our study did not detect an association of previously identified autoimmune disease risk SNPs (rs2358817 within *VTCN1* gene; rs1049550 within *ANXA11* gene; rs6679356 within *IL12RB2* gene andrs9865818 within *LPP* gene) with T1DM in Croatian T1DM trio families. However, a novel association of *IL12RB2* rs6679356 genetic variant with the early age of T1DM onset was observed. The presence of the dominant allele C (homozygotes for the C allele and heterozygotes) at this locus decreased the median age of onset by 13 months.

The observed association of SNP rs6679356 with the age of onset may be explained by the differences in T1DM etiology/genetic predisposition related to the disease onset. The early age of T1DM onset may be considered as a phenotype extreme and patients with an early disease onset could be harbouring a larger number of susceptibility loci [19]. There is a well established worldwide trend of increased T1DM incidence in the youngest age groups. Also, the onset of T1DM has peaks within different age groups, suggesting the existence of etiologically different groups of T1DM patients with different underlying genetic profiles [20]. Several studies have indicated an association between certain genetic profiles and the T1DM age of onset [21]. Different *HLA* alleles, as well as *PTPN2*, *PTPN22* and *p53* alleles, proved to be a risk in a different age of onset subgroups of T1DM patients [21], [22], [23]. The most interesting finding was that minor alleles of three *STAT4*(signal transducer and activator of transcription 4) SNPs were already associated with susceptibility to T1DM in the early-onset subgroup, providing evidence that *STAT4* is involved in earlier disease development [24]. STAT4 protein is essential for mediating responses to IL12 in lymphocytes and regulating the differentiation of T helper cells [12]. It is possible that STAT4, as an important signaling molecule of IL12, shares biological activating pathway with IL12RB2 and could demonstrate similar influence on disease age of onset.

The first association of genetic variants at the *IL12RB2* loci with PBS, an autoimmune disease, was shown by Hirschfield et al [12]. The up-regulation of the *IL12RB2* gene is associated with a number of infectious and autoimmune diseases. The co-expression of IL12RB2 and IL12RB1proteins was shown to lead to the formation of high affinity IL12 binding sites and the reconstitution of IL12 dependent signaling. The binding of IL12 to its receptor modulates autoimmune response by evoking interferon gamma production in T helper cells, thus regulating the process of differentiation, followed by regulation of natural killer cells and T lymphocytes activity. Hirschfield et al. also found association between PBC and several SNPs of the *STAT4* gene [12].

In the last few years, several studies focused on finding a genetic overlap between different autoimmune diseases. Fung et al. tested the hypothesis of shared genetic overlap between several autoimmune complex diseases and T1DM by testing association of 17 established autoimmune disease risk SNPs with T1DM [25]. The study identified *TNFAIP3* as a new T1DM locus but also found suggestive evidence of association of *STAT3, STAT4, ERAP1* and *KIF5A* genes with T1DM [25]. The *TNFAIP3* region on chromosome 6q23 has previously been associated with RA and systemic lupus erythematosus pointing to its general role in the regulation of the immune response [26], [27], [28]. Another study analysed the association of genetic variants located within genes that belong to inflammatory pathways, with three autoimmune diseases: Crohn’s disease, RA and T1DM. Such analysis showed association of 205 SNPs and 149 genes with the development of T1DM. It also identified many shared genes across T1DM, Crohn’s disease and RA [8]. So far, 17 genes have been shown to overlap between T1DM and other immune diseases such as Crohn’s disease, CD, RA, multiple sclerosis and systemic lupus erythematosis [3]. These findings contributed to better understanding of the common genetic background and the relationships between different autoimmune diseases. To the best of our knowledge, our results show for the first time that a genetic variant previously associated with PBC also has a role in defining the age of T1DM onset.

We also identified the association of the *IL12RB2* rs6679356 genetic variant, originally associated with PBC, with the age of T1DM onset, suggesting that this gene plays a role in defining the time of disease onset. In addition, this finding also points to a partially shared genetic background between PBC and T1DM. Future investigations should focus on the development and the potential use of autoimmune disease genotyping chips that will help elucidate common as well as disease-specific genetic variants.
